# Brainstorm-DUNEuro: An integrated and user-friendly Finite Element Method for modeling electromagnetic brain activity

**DOI:** 10.1016/j.neuroimage.2022.119851

**Published:** 2023-01-01

**Authors:** Takfarinas Medani, Juan Garcia-Prieto, Francois Tadel, Marios Antonakakis, Tim Erdbrügger, Malte Höltershinken, Wayne Mead, Sophie Schrader, Anand Joshi, Christian Engwer, Carsten H. Wolters, John C. Mosher, Richard M. Leahy

**Affiliations:** a Ming Hsieh Department of Electrical and Computer Engineering, University of Southern California, Los Angeles, CA 90089, United States; b Athinoula A. Martinos Center for Biomedical Imaging, Massachusetts General Hospital, Charlestown, Massachusetts, United States; c Harvard Medical School, Boston, Massachusetts, United States; d Institute for Biomagnetism and Biosignalanalysis, University of Munster, Munster, Germany; e School of Electrical and Computer Engineering, Technical University of Crete, Greece; f Department of Neurology, McGovern Medical School, University of Texas Health Science Center at Houston, Houston, TX, United States; g Department of Applied Mathematics, University of Munster, Germany; h Otto Creutzfeldt Center for Cognitive and Behavioral Neuroscience, University of Munster, Munster, Germany

**Keywords:** Head modeling, Electrophysiology, EEG/MEG/SEEG, Finite element method, Forward model, Brainstorm, DUNEuro

## Abstract

Human brain activity generates scalp potentials (electroencephalography – EEG), intracranial potentials (iEEG), and external magnetic fields (magnetoencephalography – MEG). These electrophysiology (e-phys) signals can often be measured simultaneously for research and clinical applications. The forward problem involves modeling these signals at their sensors for a given equivalent current dipole configuration within the brain. While earlier researchers modeled the head as a simple set of isotropic spheres, today’s magnetic resonance imaging (MRI) data allow for a detailed anatomic description of brain structures and anisotropic characterization of tissue conductivities. We present a complete pipeline, integrated into the Brainstorm software, that allows users to automatically generate an individual and accurate head model based on the subject’s MRI and calculate the electromagnetic forward solution using the finite element method (FEM). The head model generation is performed by integrating the latest tools for MRI segmentation and FEM mesh generation. The final head model comprises the five main compartments: white-matter, gray-matter, CSF, skull, and scalp. The anisotropic brain conductivity model is based on the effective medium approach (EMA), which estimates anisotropic conductivity tensors from diffusion-weighted imaging (DWI) data. The FEM electromagnetic forward solution is obtained through the DUNEuro library, integrated into Brainstorm, and accessible with either a user-friendly graphical interface or scripting. With tutorials and example data sets available in an open-source format on the Brainstorm website, this integrated pipeline provides access to advanced FEM tools for electromagnetic modeling to a broader neuroscience community.

## 1. Introduction

### 1.1. Electrophysiology

Electrophysiology (e-phys) is an essential field in neuroscience and medicine dealing with the electromagnetic fields generated by the nervous system. Electroencephalography (EEG) measures the electric potential on the scalp, whereas magnetoencephalography (MEG) measures magnetic fields around the head. Collectively referred to as MEEG, these are the two most prominent non-invasive e-phys techniques (Baillet et al., 2001; Gross et al., 2021; Hämäläinen et al., 1993). Electrocorticography (ECoG) and stereo-EEG (SEEG) (collectively IEEG) are invasive measurements of potentials from surgically implanted electrodes in the brain. They are all complementary techniques characterized by high temporal resolution and sufficient spatial accuracy to help in understanding the link between behavior and neural activity and, importantly, help in treating neurological disorders such as epilepsy or major depression, and neurodegenerative diseases such as Parkinson’s or Alzheimer’s disease.

### 1.2. E-phys forward and inverse modeling

To improve our capacity to interpret an electrophysiology recording, we can estimate the neuronal activity underlying the measured data. This implies solving: first the forward and then the inverse problem. The e-phys forward problem involves modeling the signal at the MEEG/IEEG sensors generated from modeled neuronal sources placed within the brain. In contrast, the inverse problem identifies and localizes the active brain sources that generate the observed e-phys data (Baillet et al., 2001; Brette and Destexhe, 2012; Gross et al., 2021; Hämäläinen et al., 1993; Mosher et al., 1992; Salmelin and Baillet, 2009). The accuracy of inverse solution, which can be classified into focal current modeling, beamforming and distributed current modeling (Gross et al., 2021), depends not only on noise characteristics and the properties of the inversion method itself, but also on the accuracy of the forward solution. Therefore, the head model, also referred to as the volume conductor model, needs to reflect the real head geometry and the conductive properties of its tissues as closely as possible.

A commonly used head model assumes a set of nested concentric overlapping spheres, for which the (nearly) analytical solution exists (Baillet et al., 2001; Brette and Destexhe, 2012; Mosher et al., 1999); however, many studies have shown that differences between a spherical shape and a realistic geometry can create localization errors (Leahy et al., 1998; Vorwerk et al., 2014). Additionally, isotropic conductivity is commonly associated with each tissue compartment, although brain tissues, especially white matter, exhibit significant anisotropy (Drechsler et al., 2022, 2009; Güllmar et al., 2010; Vorwerk et al., 2014; Wolters et al., 2006, 2001). It has been shown that the more anisotropy there is between sources and sensors, the greater the impact on modeling accuracy (Drechsler et al., 2022; Güllmar et al., 2010; Wolters et al., 2006). Furthermore, Güllmar et al. (2010) have shown that white matter anisotropy might cause significant changes in source orientation and magnitude, while having less effect on source localization. The orientation of dipoles may also help in identification of the sulcal wall in which epileptic activity occurs (Salayev et al., 2006). Therefore, realistic head modeling, with individualized geometry and anisotropic tissue conductivity, is a critical step for accurate e-phys modeling.

Magnetic resonance images (MRI) are now a standard modality to build realistic individualized head models. A combination of T1w- and T2w-MRI enables an accurate segmentation of the scalp, skull, cerebrospinal fluid (CSF), brain gray and white matter (Antonakakis et al., 2020, 2019). Due to the special importance of the low-conducting skull for the EEG (Montes-Restrepo et al., 2014; Vorwerk et al., 2019a) and the high contrast in the T2w-MRI between CSF and skull, an additional T2w-MRI specifically allows an improved segmentation of the inner skull surface. This bi-modal MRI dataset even enables distinguishing between compacta and spongiosa, thus modeling the three layers of the skull (Antonakakis et al., 2020; Dannhauer et al., 2011; Montes-Restrepo et al., 2014). Finally, diffusion-weighted imaging (DWI) and an effective medium approach for relating the diffusion tensors to conductivity tensors (Lee et al., 2006; Marino et al., 2021; Tuch et al., 2001; Wang et al., 2008) can be used to estimate anisotropic conductivity in brain tissue.

A large number of numerical approaches for solving the forward problem using a realistic head shape have been investigated in the literature, including the boundary element methods (BEMs) (Gramfort et al., 2011; Mosher et al., 1999), the BEM fast multipole method (BEM-FMM)(Makarov et al., 2021), finite volume methods (Cook and Koles, 2006), finite difference methods (FDMs) (Vatta et al., 2010) and finite element methods (FEMs) (Pursiainen et al., 2011; Schimpf et al., 2002; Tanzer et al., 2005; Van Uitert et al., 2004; Vermaas et al., 2020; Weinstein et al., 2000; Wolters et al., 2007; Yan et al., 1991). While all represent anatomical boundaries more realistically than a spherical model, the FDM and FEM methods can propitiate a higher accuracy because they can model both inhomogeneities and anisotropy in brain tissue. For this reason, in this work, our focus is on the FEM approach.

FEM is a computational approach based on the discretization of the volume in a series of finite mesh elements (Strang and Fix, 1974). Initially developed in the context of fluid dynamics and structural analysis, FEM has attracted much attention for modeling the e-phys forward problem (Beltrachini, 2019a, 2019b; Marin et al., 1998; Medani et al., 2015; 2014; Pursiainen et al., 2011; Rullmann et al., 2009; Vallaghé and Papadopoulo, 2010; Wolters, 2003; Wolters et al., 2007).

### 1.3. E-phys modeling tools

Several research software^1^ tools have been developed to obtain the forward solution for the e-phys problem (Baillet et al., 2010), including Brainstorm (Tadel et al., 2011), SPM (Friston, 2004), MNE (Gramfort et al., 2014), EEGLAB-NFT (Akalin Acar and Makeig, 2008), Fieldtrip (Oostenveld et al., 2011), OpenMEEG (Gramfort et al., 2011), the Helsinki BEM Framework LCISA(Matti Stenroos, 2016), the sLORETA package (Pascual-Marqui R D, 2002), PyEEG (Sheng Bao et al., 2011), Biosig (Vidaurre et al., 2011), and NUTMEG (Dalal et al., 2011). These tools offer different combinations of methods and implementations at different levels of accuracy for the forward solution, with emphasis on spherical or BEM models. FEM modeling is provided by Sim-Bio,^2^ Zeffiro (He et al., 2020), and DUNEuro (Schrader et al., 2021). The Fieldtrip-SimBio integration (Vorwerk et al., 2018) provides only EEG forward modeling through MATLAB scripting, while Zeffiro, SimBio and DUNEuro also incorporate MEG modeling.

Even with such an extensive list of advanced tools, a complete multiplatform e-phys FEM package providing an easy-to-use interface has been unavailable up to this point. The software pipeline introduced in this paper is designed to address this gap, facilitating use of FEM through a guided user interface (GUI) and through scripts for advanced users, producing a complete forward-modeling pipeline integrated within the Brainstorm analysis software. The primary software tools on which we base this development are DUNEuro and Brainstorm:

*DUNEuro* is an open-source software library for solving forward problems in bioelectromagnetism using modern FEM approaches. It is based on the *DUNE*^3^ computational library (Bastian et al., 2021, 2008). DUNEuro provides an extendible framework for using advanced FEM in the context of e-phys applications (Schrader et al., 2021). DUNEuro is implemented using C++ with limited external dependencies and provides interfaces to Python and MATLAB. The source code of DUNEuro is free and publicly accessible under GNU GPL.^4^ A tutorial that describes the low-level interface when computing MEEG leadfields is available on the DUNEuro website.^5^ Importantly, DUNEuro offers modern FEM methods, such as fitted CG and DG, as well as unfitted FEM (UDG and CutFEM), with a variety of FEM source models. The DUNEuro solver, however, can be used only by scripting, and its use is limited to Linux users.

*Brainstorm* is free and open-source software^6^ designed to model and analyze e-phys data (Tadel et al., 2019, 2011). Brainstorm is designed to be used in interactive and scripted settings for automated data analysis and visualization. Brainstorm is available for all three major computer platforms through the MATLAB framework, including a standalone version that requires only the free MATLAB Runtime environment. Brainstorm offers several methods for solving the forward problem, including simple spherical head models (de Munck and Peters, 1993; Zhang, 1995) and overlapping spheres (Huang et al., 1999). It also offers BEM solutions through the OpenMEEG package that can be used either for MEEG or IEEG, i.e. non-invasive and invasive models. By additionally integrating the DUNEuro library into Brainstorm, we intend to provide the broad neuroscientific community easy access to analysis of e-phys data using FEM.

FEM based approaches require preparation of the volume conductor model. In practical terms, researchers adopting the FEM approach must discretize the volume of the head into a finite number of small elements and assign an associated conductivity to each. To generate a realistic FEM head model, MRI images are segmented into different tissue types, from which FEM volume elements are generated using a pipeline of various methods and toolboxes. To make this work complete, we have added a set of tools to automate volume segmentation and discretization from the subject MRI. Generation of the anisotropic volume conductor is included as an additional automatic process to compute anisotropic conductivity from DWI data.

The pipeline presented in this paper aggregates Brainstorm with a complete FEM package for subject-based e-phys forward modeling through a GUI. The pipeline input is the MRI scan of the subject. The final output consists of interactive visualization of the segmented head, the tessellated volumes, and the conductivity tensor, as well as the final leadfield matrix. The latter can be used with the different inverse methods such as minimum norm (Baillet et al., 2001), dSPM (Dale et al., 2000), sLORETA (Pascual-Marqui R D, 2002) and LCMV (Jaiswal et al., 2020), which are all included within Brainstorm for source localization.^7^

In the following, we first present the FEM workflow within Brainstorm with a complete description of the methodology and the tools used to build this pipeline. We exclude from this text a detailed description of the FEM methodology itself (the references we include provide this information). On the other hand, computation of the conductivity tensor based on the DWI images is a step which has been implemented here from scratch and we describe it in more detail. We then demonstrate the Brainstorm FEM pipeline in processing real MEEG data, including generation of the complete head model and computation of the leadfield. Finally, we demonstrate application to source reconstruction for a sample set of somatosensory MEEG evoked response data.

## 2. Brainstorm-FEM workflow

The main workflow of the Brainstorm-FEM pipeline is divided into two phases. The first phase generates the finite element volume conductor, including the head geometry and the tissue conductivity. The second phase uses the volume conductor model to solve the forward problem and compute the leadfield matrix.

### 2.1. Phase one: volume conductor modeling

The finite element (FE) volume conductor is a description model of the geometry and conductivity of the head tissues using FE nodes (vertices) connected by FE elements (tetrahedrons or hexahedrons), where each volume element is associated with the conductivity of its respective tissue. Scalar or tensor values are assigned respectively for isotropic and anisotropic conductivities.

**Fig. 1.**
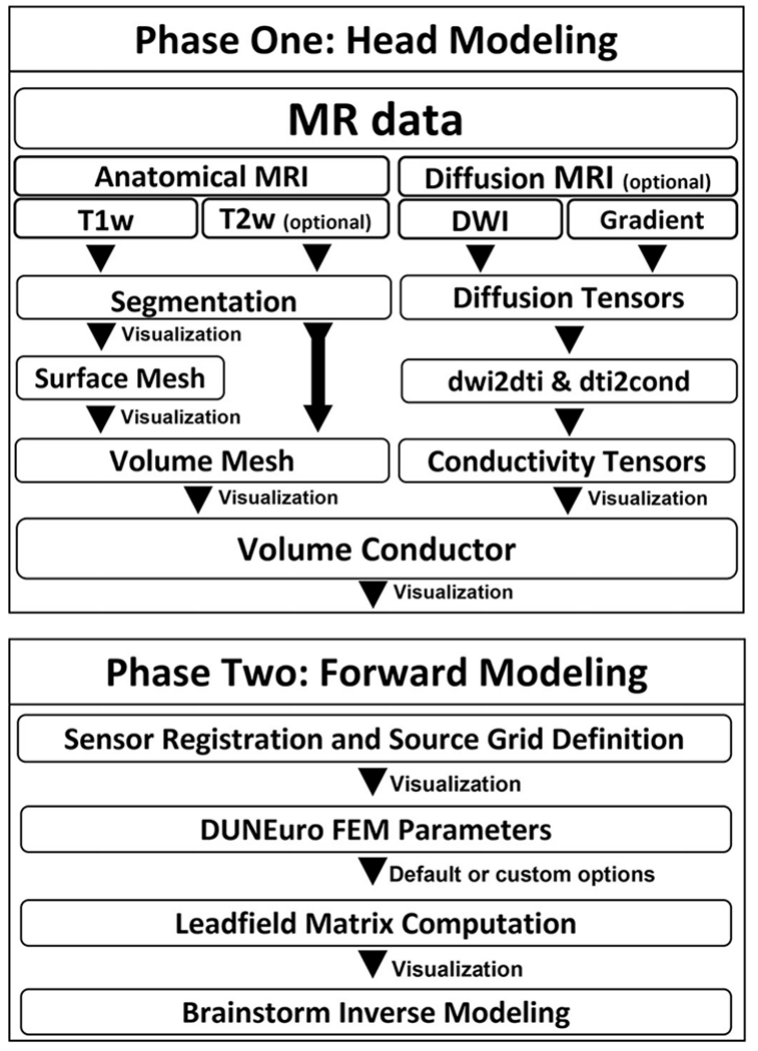
Overview of the Brainstorm-FEM workflow. Top: “Phase One: Head modeling. Bottom: “Phase two: Forward modeling”.

The process of volume mesh generation includes three main steps, as shown in Fig. 1: (1) MRI segmentation, (2) surface tessellation or meshing, and (3) volume tessellation. Brainstorm can handle all three steps; furthermore, users can import pre-processed data from third-party software at any of these steps (segmentation, surface mesh, or volume mesh).

#### Segmentation:

Segmenting an MRI scan is the assignment of each voxel in the MRI into a specific tissue class. For MRI processing and segmentation, Brainstorm is fully interfaced with well-known software such as FastSurfer (Henschel et al., 2020), FreeSurfer (Dale et al., 1999), SPM (Ashburner and Friston, 2005), CAT12 (Gaser and Dahnke, 2016), Brain-Suite (Shattuck and Leahy, 2002), CIVET (Ad-Dab’bagh et al., 2006), and BrainVISA (Rivière et al., 2003). Note that only a T1-weighted (T1w) image is required for the complete head segmentation, although T2-weighted (Tw2) data is optional but strongly recommended for better definition of the interface between skull and cerebrospinal fluid (CSF). The typical full head segmentation includes the five main tissues: white matter (WM), gray matter (GM), CSF, skull, and scalp. Brainstorm allows the import of segmentation results from the previously listed software or from other software provided a supported input format in used.

#### Surface Mesh:

The surfaces separating different tissues are represented by sets of triangles forming a mesh. The vertices of each triangle within each surface are defined in relation to the segmented voxels described in the previous step.

Surface meshes generated from external software, including Brain-Suite, FSL, Curry, and BrainVISA can also be loaded into Brainstorm. Alternatively, Brainstorm can generate simpler surfaces (scalp, outer skull, inner skull) directly from an MRI. These surfaces can be used for BEM^8^ forward modeling computation but are not suitable for use with the FEM methods described here.

#### Volume Mesh:

the volume mesh is generated by filling each tissue region in between surfaces with FE volume elements. Brainstorm supports both voxel-based and surface-based meshes. Voxel-based meshes are obtained from the conversion of each segmented voxel into a hexahedron or tetrahedron. Brainstorm calls the FieldTrip toolbox for a hexahedral mesh element or the Brain2Mesh toolbox for a tetrahedral mesh. For the surface-based mesh, Brainstorm uses the Iso2Mesh toolbox (Fang and Boas, 2009; Tran et al., 2020) to create a tetrahedral mesh from the nested surfaces. Additionally, users can also import FE meshes from other software tools including Gmsh, SimNibs, FieldTrip, and Roast, or other tools whose output can be converted into a Brainstorm supported format.

As an alternative to importing these external software results, Brainstorm provides packaged routines that integrate all the above steps using tools including Iso2Mesh, SimNibs, Roast, Brain2Mesh, and Fieldtrip. Below is a brief description of these integrated tools; readers can also refer to the detailed online tutorial^9^ on the Brainstorm website.

### 2.2. Tools for finite element (FE) mesh generation

Brainstorm packaged routines are called “plug-ins”. Several are provided to generate the FEM mesh,^10^ including the following packages. Brainstorm automatically downloads and installs all dependency toolboxes when required:

#### Iso2mesh:

The software iso2mesh is a Matlab toolbox (Fang and Boas, 2009) that can be used to generate a tetrahedral mesh from nested surfaces or segmented tissues. This toolbox is integrated into many other tools, such also Roast (Huang et al., 2019) and Brain2Mesh (Tran et al., 2020).

#### SimNIBS:

SimNibs is software dedicated to TES/TMS simulation using FEM. Brainstorm calls the SimNIBS-*headreco* process for FEM head reconstruction (Saturnino et al., 2019). To use this method, the SimNibs software needs to be installed in the user operating systems. SimNIBS *headreco* uses the SPM12 and CAT12 toolboxes (Gaser and Dahnke, 2016) (automatically installed by Brainstorm, when needed) for segmenting MRI scans; then the tissue maps are cleaned and used to create a surface tessellation. Finally, the FE mesh is generated by forming tetrahedrons between tissue surfaces using Gmsh (Geuzaine and Remacle, 2009). Currently, SimNIBS is the recommended option for realistic head model generation.

#### Roast:

Roast is a MATLAB toolbox designed for TES simulation using the FEM (Huang et al., 2019). Brainstorm can internally call the Roast toolbox for FE head model generation. Roast uses SPM to segment the MRI and then calls Iso2mesh to generate a tetrahedral mesh. The Roast output mesh is voxel-based, whereas the SimNIBS headreco output is surface-based. Roast is available as a Brainstorm plugin and is downloaded automatically when required.

#### Brain2mesh:

Brain2mesh is a MATLAB toolbox for FE mesh generation. Brain2mesh is available as a Brainstorm plugin and is downloaded automatically when required. To generate a head model with Brain2Mesh, Brainstorm first calls SPM12 for MRI tissue segmentation, followed by Brain2mesh routines for tetrahedral mesh generation.

#### FieldTrip:

**Fig. 2.**
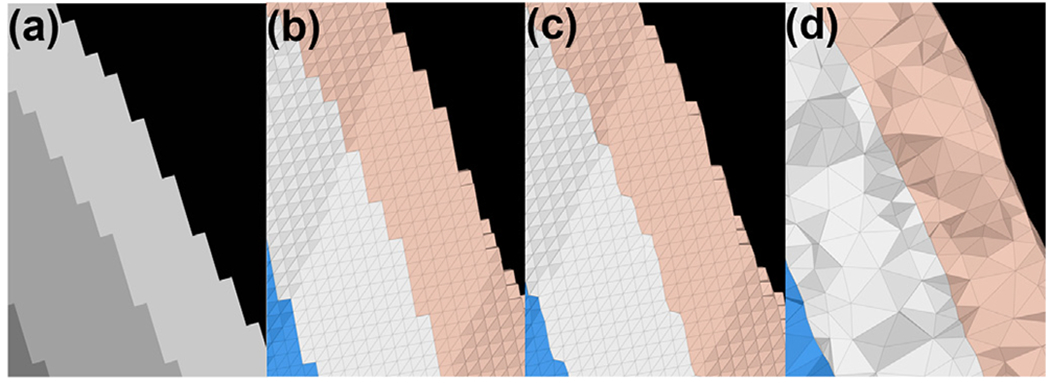
Illustration of different tessellation schemes: (a) Segmented MRI, view at the interfaces of the scalp, skull, and CSF. (b) The voxel-based hexahedral mesh. (c) The voxel-based adapted hexahedral mesh with node shift at the interfaces.(d) The surface-based tetrahedral mesh.

This package is implemented using the Fieldtrip toolbox (Oostenveld et al., 2011), which includes MRI segmentation with SPM, from which both the surface tessellation and volume mesh can be generated. Both hexahedral and tetrahedral meshes can be obtained; furthermore, an adaptive hexahedral mesh from the segmentation can also be obtained, as shown in Fig. 2. This implementation is achieved by integration of the FieldTrip-SimBio (Vorwerk et al., 2018) package and calling the functions *ft_volumesegment*^11^ and *ft_prepare_mesh*.^12^

Note that these research tools do not generally support the generation of head models for subjects with prior brain resections, tumors, stroke, or other pathology. In these cases, segmentations will often need to be manually edited before mesh generation. Documentation on the effect of mesh quality and mesh resolution are available on the brainstorm website.^13^

### 2.3. Phase one: conductivity tensors (optional)

The default assumption is that all tissue types are isotropic, and one scalar value is assigned for each. Brainstorm assigns the standard values by default as follows: 0.43 S/m for the scalp, 0.01 S/m for the skull, 1.79 S/m for the CSF, 0.33 S/m for the GM, and 0.14 S/m for the WM (Vorwerk et al., 2014). The user can change these scalar values. Note that higher default skull conductivity values have also been discussed (Dannhauer et al., 2011) and it is also known that this important parameter might be inter- and intra-individually varying, due to age for example (Antonakakis et al., 2020). Because of the special importance of skull conductivity to EEG source analysis (Vorwerk et al., 2019a), if MR image quality allows, we recommend distinguishing between compact and spongy bone and modeling these skull compartments explicitly by assigning each voxel to one of the two conductivities as suggested in (Dannhauer et al., 2011; Lanfer et al., 2012). In some cases it might be valuable to estimate individual conductivities, which can be done for example by a skull-conductivity calibration procedure as suggested by (Antonakakis et al., 2020; Schrader et al., 2020), although currently this option is not available within Brainstorm.

The approach described here allows optional inclusion of a three-by-three matrix (second-rank tensor) that represents anisotropic conductivity in each finite element. We adopt the most commonly used approach to deriving brain tissue anisotropy from DWI data: “the effective medium approach (EMA)” (Güllmar et al., 2010; Haueisen et al., 2002; Lee et al., 2006; Marino et al., 2021; Rullmann et al., 2009; Tuch et al., 2001; Vorwerk et al., 2014; Wang et al., 2008; Wolters et al., 2006). The EMA assumes that the electrical conductivity tensor σ and the water diffusion tensor *d* share the same eigenvectors with the eigenvalues linearly scaled by a known factor *s*,

(1) σ=sd

The value of *s* is fixed to *0.736 S-s/mm^3^* (Siemens-seconds/millimeter^3^) as in (Haueisen et al., 2002; Tuch et al., 2001). An EMA with a volume constraint has also been proposed (EMA+VC), where the geometric mean of the eigenvalues is retained as its equivalent isotropic value σ^iso^ (Rullmann et al., 2009; Wolters, 2003). The scaling factor in this case is computed empirically adopting the approach used in (Vorwerk et al., 2014),

(2) $$s=\sigma^{iso}{\left(D_{wm}/N_{wm}\right)}^{-1}$$

where *D_wm_* is the sum over all white matter voxels of the 3rd root of the product of the three diffusion tensor eigenvalues and *N_wm_* is the number of voxels. The final conductivity tensors are then assigned at the barycenter of each FEM element.

#### Realistic tensor:

**Fig. 3.**
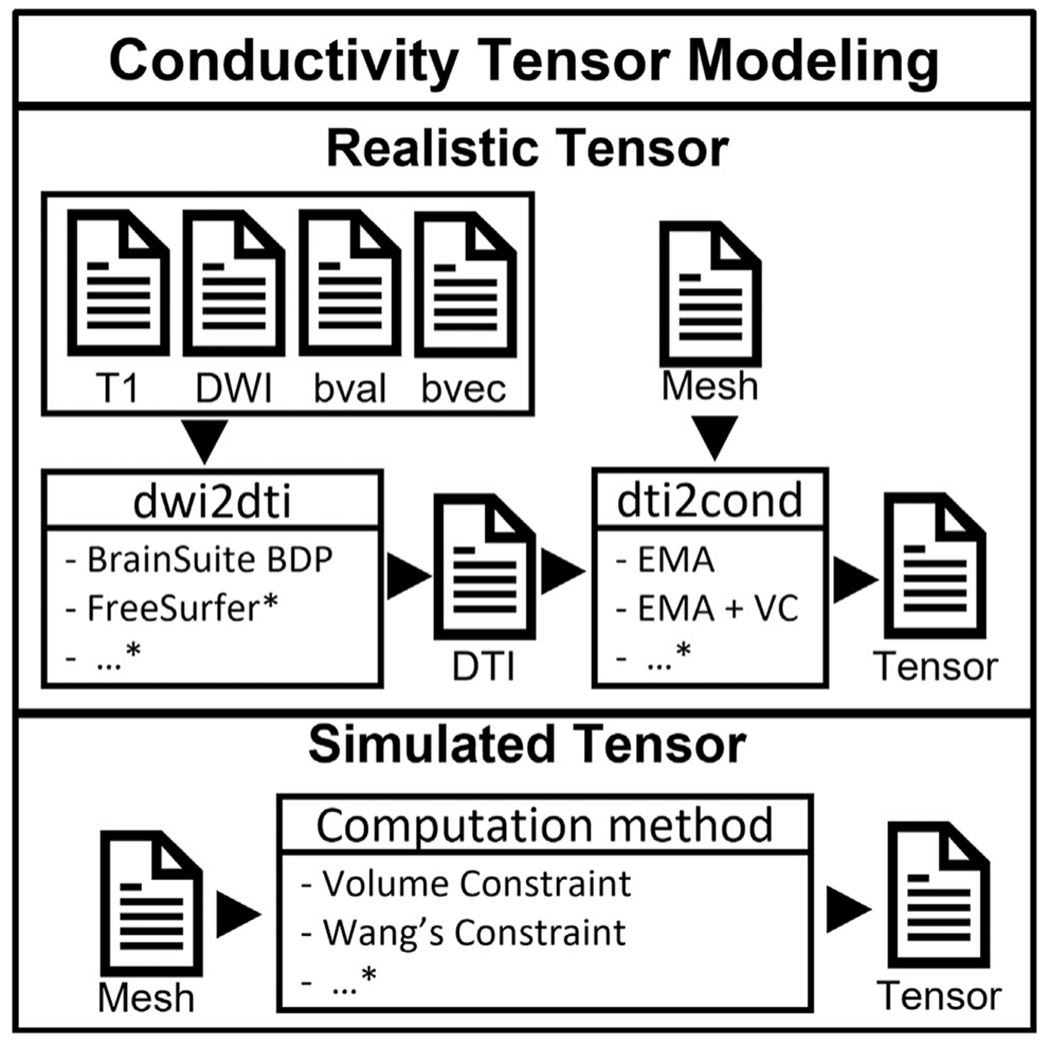
Schematic view of the FEM tensor generation process within Brainstorm. Realistic tensor modeling requires subject DWI data. The * refers to methods not implemented and the possibility to import data from third-party software.

The conductivity tensors are estimated from the DWI data. This process is completed in two steps, as shown in Fig. 3. The first step, ***“dwi2dti”***,^14^ maps DWI data to a diffusion tensor image (DTI). The second step, ***“dti2cond”*,** maps the DTI to conductivity tensors. The first step can be performed externally using any third-party software (Soares et al., 2013) such as FSL, DTIStudio, FreeSurfer, or SPM-HySco (Ruthotto et al., 2012). Here we used the BrainSuite^15^ software (Shattuck and Leahy, 2002), which can be called internally from Brainstorm. Briefly, the following stages are executed: First, the DWI is co-registered to the T1w space, then BrainSuite skull stripping (BSE) is used to remove non-brain tissues, after that the BrainSuite bias field correction (BFC) is applied. Finally, the BrainSuite Diffusion Pipeline (BDP) is executed to compute the DTI. Other packages should follow a similar workflow; users can then import the resulting DTI data after conversion to the Brainstorm format. Finally, the process ***“dti2cond”***,^16^ converts the DTI to electrical conductivity, which is completed using Brainstorm’s built-in pipeline editor. In this step, each MRI voxel is mapped to its corresponding FE mesh elements, and the conversion is applied. Two approaches are integrated in Brainstorm: direct mapping ‘EMA’ and ‘EMA+VC’ as described above (Güllmar et al., 2010; Rullmann et al., 2009; Saturnino et al., 2019; Vorwerk et al., 2014). The constrained approach prevents the problem of high conductivity values that can appear when a direct mapping is used. For both methods, to ensure that all the conductivity values remain realistic, a maximal conductivity of 2 S/m is fixed, and a maximal ratio of 10 between the longitudinal and transversal eigenvalues is enforced (Saturnino et al., 2019). Note that realistic tensors are estimated from the DWI only for brain tissue.

#### Simulated tensor:

This option can be useful to build custom anisotropy models for test and research scenarios. Users can create a tensor on the desired tissue by setting a scalar ratio between longitudinal and transversal orientation (for the skull layer for example). Supported methods include the volume constrained approach (Wolters et al., 2006). Advanced users can also implement their own methods and integrate them as a *Brainstorm process*.^17^

The result of Phase One is the finite element volume conductor, which combines both the geometry and the conductivity information of the major head tissues. The computation time for this phase can vary from 1 h to 4 h depending on the selected method, the mesh resolution, and the performance of the computer.^18^With the volume conductor geometry and conductivity as well as source model and sensor locations defined the forward solution can then be computed as explained in phase two.

### 2.4. Phase two: FEM forward modeling

This second phase uses the volume conductor model obtained in the previous phase to compute a forward model to relate sources and sensors. There are two steps; the first step requires the positions of the sensors and the source grid (source space). The second step specifies the parameters of the FEM calculation in DUNEuro options and computes the forward model. The final output of this process is the “leadfield” matrix, yielding the transfer function that relates each source to each sensor.

#### Sensor model registration:

Brainstorm offers many interactive functionalities that facilitate the placement of sensors relative to the selected head model. For MEG recordings, sensor positions are usually read directly from the vendor recordings. For EEG scalp electrodes, Brainstorm offers a large panel of sensor templates that can be adapted to any head model, or users can load their digitized cap location data. For ECoG and sEEG, cortical and subcortical contact locations can be imported or positioned interactively on a post-implantation CT, MRI or on the head model.

#### Source grid definition:

The source grid, or source space, is a set of points at which current dipoles are placed to model neuronal activity in the brain. Brainstorm offers three options to define the source space: the cortical surface, the MRI volume, and a custom source model where users define their own set of dipoles. Note that currently, placement of equivalent current dipoles within anisotropic gray matter tissues is not recommended in standard adult EEG and MEG source analysis scenarios. Further investigations on the accuracy of FEM source models within anisotropic tissue are still under investigation (Drechsler et al., 2022).

#### Leadfield computation:

**Fig. 4.**
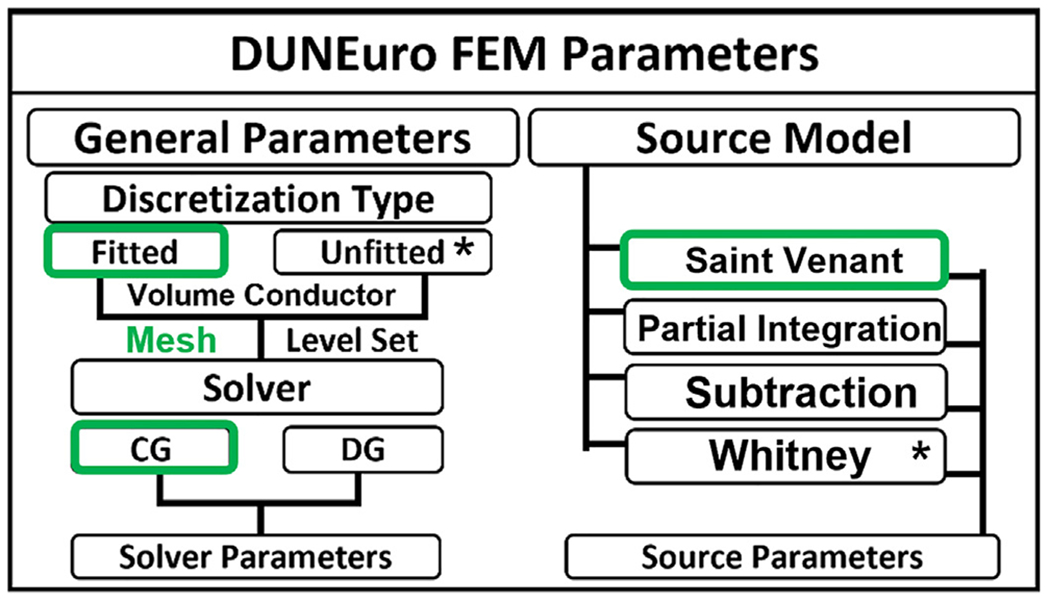
Summary of Brainstorm-DUNEuro parameters. Left are the general FEM parameters related to the discretization methods and the solvers. Right is the list of FEM source models with the choice of parameters. The * refers to the method that are available through the DUNEuro interface.

The DUNEuro-FEM computation within Brainstorm follows a guided process similar to other previously implemented head model methods, such as OpenMEEG-BEM. The DUNEuro-FEM parameters can be tuned by the user either from the GUI interface or from scripts. A simplified list of available parameters is listed in Fig. 4; the complete list can be found on the Brainstorm and DUNEuro websites.

Several FEM variations have been proposed such as Lagrange or continuous Galerkin approaches (CG) (Güllmar et al., 2010; Medani et al., 2015; Rullmann et al., 2009; Vallaghé and Papadopoulo, 2010; Vorwerk et al., 2014; Wolters, 2003; Wolters et al., 2007). Recently, the discontinuous Galerkin FEM (DG) was proposed for the EEG forward problem (Engwer et al., 2017; Nüßing et al., 2016; Piastra et al., 2018). Furthermore, new “unfitted” FEMs, namely CutFEM (Erdbrügger et al., 2022; NüßRing, 2018; Schrader et al., 2021) and the unfitted discontinuous Galerkin (UDG) (Nüßing et al., 2016) methods have recently been introduced to solve the MEEG forward problem. All these methods are available within the DUNEuro library. In addition to the various variational approaches mentioned above, several FEM source modeling approaches have also been investigated (Drechsler et al., 2009; Lew et al., 2009, 2007; Medani et al., 2015). The most commonly known source models are the Saint Venant, the partial integration (PI), and the subtraction model. Alternative new source models are under development such as the Whitney and multipole approaches (Pursiainen et al., 2016; Vorwerk et al., 2019b) and, most recently, localized subtraction (Höltershinken et al., 2022).

Once the FEM computation is completed, the leadfield matrix is then loaded to the Brainstorm database and can be used for further advanced analysis and visualization (see Fig. 10). The computation time of this phase depends on several parameters such as the discretization type, the resolution of the mesh, the source model, the number of sensors as well as on the selected modality (EEG/MEG).

### 2.5. Brainstorm-DUNEuro interface

**Fig. 5.**
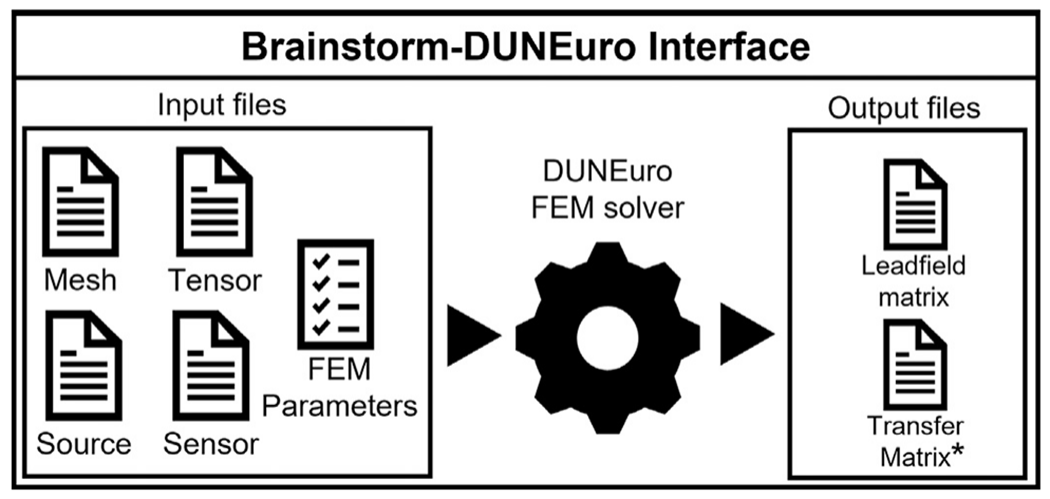
Schematic of the Brainstorm-DUNEuro interface showing input and output files.

The Brainstorm-DUNEuro interface is a module written in MATLAB and fully integrated into the Brainstorm source code. The interface includes a set of functions that convert Brainstorm data and parameters into input files that are used by DUNEuro, which in turn generates output files that are returned to Brainstorm, as presented in Fig. 5. The FEM user interface includes several parameters. The full list and all available methods can be found on the Brainstorm and DUNEuro webpages. For each option, Brainstorm defaults to the values recommended by the DUNEuro developers; however advanced users can change these parameters:

#### General FEM parameters:

These parameters are related to the FEM discretization methods and the solver options as highlighted in Fig. 4. The current interface supports the fitted FEM discretization scheme, and both the Continuous Galerkin (CG) and the Discontinuous Galerkin (DG) approaches (Engwer et al., 2017; Nüßing et al., 2016; Piastra et al., 2018). Unfitted approaches will be made available as soon as possible.

#### Source model parameters:

As mentioned in the previous section, several FEM dipole-models have been investigated in the literature. Most of these models are implemented within DUNEuro. Brainstorm allows users to choose between them. The Saint-Venant, the subtraction, and the partial integration (PI) models are currently available, as shown in Fig. 4. The Whitney and localized subtraction approaches will also be implemented in future releases.

#### DUNEuro FEM solver:

Since DUNEuro was initially developed for Linux-based operating systems, no Windows versions are directly available. By incorporating DUNEuro into Brainstorm we have made it accessible under the Windows and Mac OS. Detailed documentation regarding the cross-compilation is available in the Brainstorm GitHub repository.^19^ There is no action required from the users’ perspective for DUNEuro compilation and installation. Brainstorm handles the installation process and the DUNEuro binaries are automatically downloaded when required. When the FEM computation is performed within Brainstorm, the following steps are executed internally (see Fig. 5):

- Write the mesh file, which contain the lists of vertices and elements.
- Write the conductivity tensor file, which contains tissue conductivity tensor values.
- Write the source file, which contains the coordinates, and the orientations of the dipoles.
- Write the sensor file, which contains the coordinates of the sensors (and the orientations for MEG sensors).
- Write the Brainstorm-DUNEuro FEM parameters.
- Call the DUNEuro solver to compute the FEM transfer matrix and the FEM leadfield.

Once the FEM computation is complete, two files are generated: the leadfield matrix file, which is then loaded to the Brainstorm database, and optionally the transfer matrix (Wolters et al., 2004) file which can be used for further advanced analysis. These steps are executed internally and transparently to the user. Moreover, these steps are also available for scripted pipelines through specific Brainstorm processes (as described in web-based tutorials^20^).

### 2.6. FEM processing tools

To facilitate manipulation of the finite mesh and associated conductivities, additional functionalities are integrated into the Brainstorm GUI, such as resection of the mesh to remove unnecessary parts of the head (such as neck and shoulders), which can speed-up FEM computation without loss of accuracy (Lanfer et al., 2012). Moreover, the resection option also allows “defacing” of a subject’s face mesh to aid de-identification. Additionally, functions to export/import the FE mesh are also integrated, allowing the users more flexibility and the ability to interface with other software.

#### Mesh visualization:

**Fig. 6.**
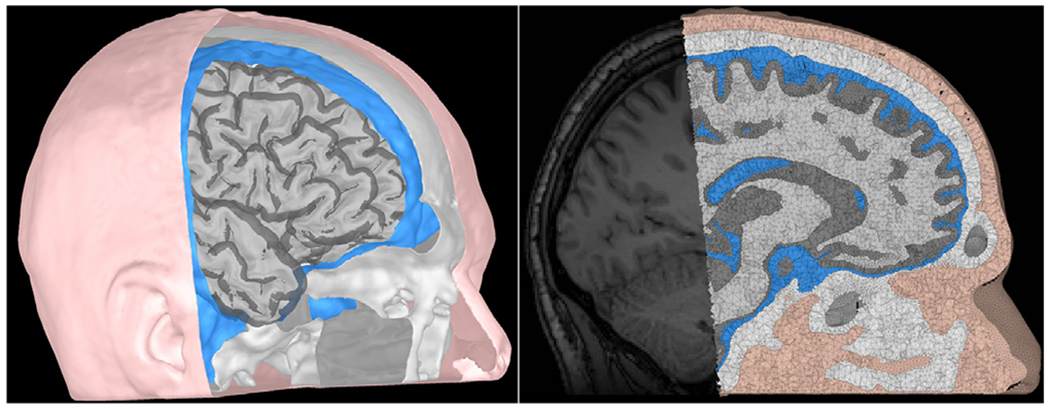
Left: surface head model; tissues from inner to outer: wm, gm, CSF, skull, and scalp. Right: Overlay of the tetrahedral volume mesh on the MRI.

Brainstorm already has multiple visualization modules, including the MRI viewer and rendering of head surfaces, cortex, and sensors. In the current release, users can display and overlay the FE mesh on the subject MRI for visual inspection of the quality of the mesh (see Fig. 6).

#### Tensor visualization:

**Fig. 7.**
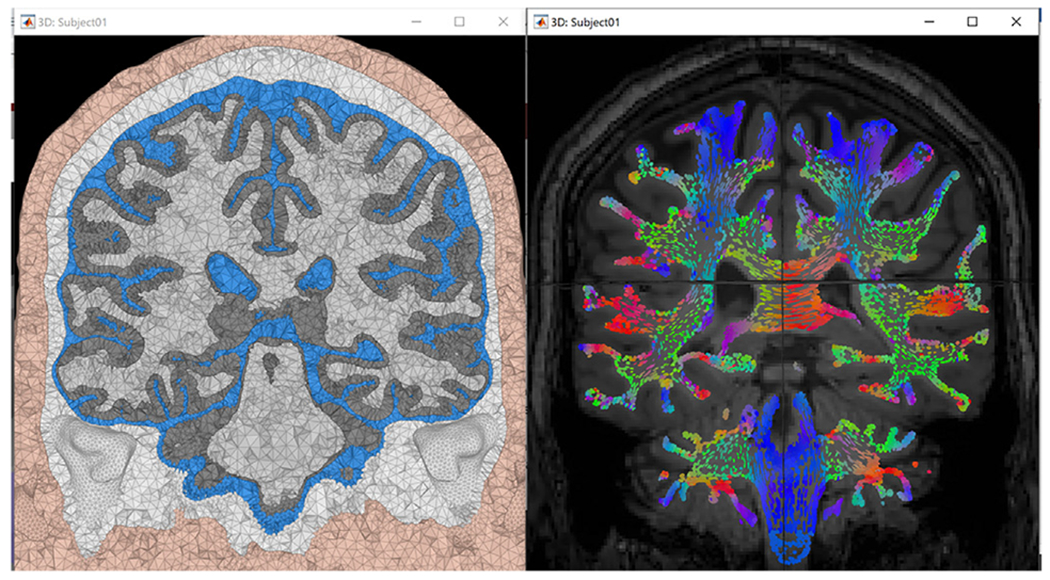
Visualization of the realistic FEM volume conductor with Brainstorm. Left: The tetrahedral volume mesh with five compartments: wm, gm, CSF, skull, and scalp. Right: The conductivity tensors in wm, overlaid on the subject’s MRI, as color-coded ellipsoids computed from the DWI data: red for dominant eigenvector oriented right-left, green for anterior-posterior, and blue for superior-inferior.

When the conductivity tensors are computed and saved to the database, users can display the tensor either as ellipsoids or as lines oriented along the main eigenvector direction. The tensor display can be overlaid on the FEM mesh, MRI, or both. The orientation is color-coded as indicated in Fig. 7, which shows examples of the tensors displayed on the subject MRI.

#### Leadfield visualization:

The result of the forward computation is the “leadfield matrix,” where the number of rows equals the number of sensor channels, and the number of columns equals the number of elemental sources, such that we have a mapping of every cortical dipole to every sensor. With the new package, users can also display the leadfield vectors sampled on the source space positions (see Fig. 10).

## 3. Application

### 3.1. Data description

To demonstrate the functionality of the new FEM pipeline, publicly available data, including MRI (T1w, T2w, and DWI) and MEEG, from a healthy adult subject were used. These data and a step-by-step tutorial for processing the results presented here can be found on the Brainstorm website.^21^

The subject underwent MRI scanning (Philips Medical Systems), providing a T1w image (3T field strength, flip angle 8°, TR 7.9 s, dimensions 512 × 512 × 200, and voxel dimensions of 0.469 × 0.469 × 0.939 mm), and a T2w spin-echo image (3T field strength, 78.57% phase FOV, 90° flip angle, SAR 0.327, voxel dimensions 560 × 560 × 55, dimensions of 0.429 × 0.429 × 3 mm ) and DWI sequences (voxel dimensions 512×512×200 and dimensions of 0.469 × 0.469 × 0.939 mm, diffusion-sensitizing gradients in 32 non-collinear directions). Somatosensory evoked potentials (EEG-SEP) and fields (MEG-SEF) were simultaneously recorded by stimulating the median nerve at the wrist of the left arm with monophasic square-wave electrical pulses of 0.2 ms duration. The interstimulus interval was 500 ms. The intensity was adjusted to invoke an apparent movement of the thumb while remaining within the comfort zone for the subject. The data were acquired with a sampling rate of 1 kHz. The EEG was measured using a 74-channel cap (EASYCAP GmbH, Herrsching, Germany). Electrode positions were digitized using a Polhemus device (FASTRAK, Polhemus Incorporated, Colchester, Vermont, U.S.A.). The reference was placed on the FCz channel. MEG recordings covering the whole head with 204 planar gradiometers and 102 magnetometers were acquired using an Elekta Triux (Megin, Finland) scanner.

This data, similar to the WWU DUNEuro reference data (Piastra et al., 2020), is well suited for demonstration purposes since the early response around 20 ms post-stimulus has been extensively studied (Antonakakis et al., 2019; Buchner et al., 1995), indicating a single focal and quasi-tangentially oriented dipolar source in Brodmann area 3b. The main purpose here is to show a practical case using the presented pipeline with available data that users can reproduce.

### 3.2. Phase one: head modeling

The SimNibs process was used in this example to generate the volume mesh and a cortical surface (source) model. Both T1w and T2w images were used to ensure good tissue segmentation, as recommended in the previous section. White matter anisotropy was estimated from the DWI using Brainsuite and by applying the “EMA+VC” method. Standard isotropic conductivities were assigned to the remaining tissues. Fig. 7 shows a view of the resulting model.

### 3.3. Phase two: forward modeling

#### EEG/MEG data processing:

**Fig. 8.**
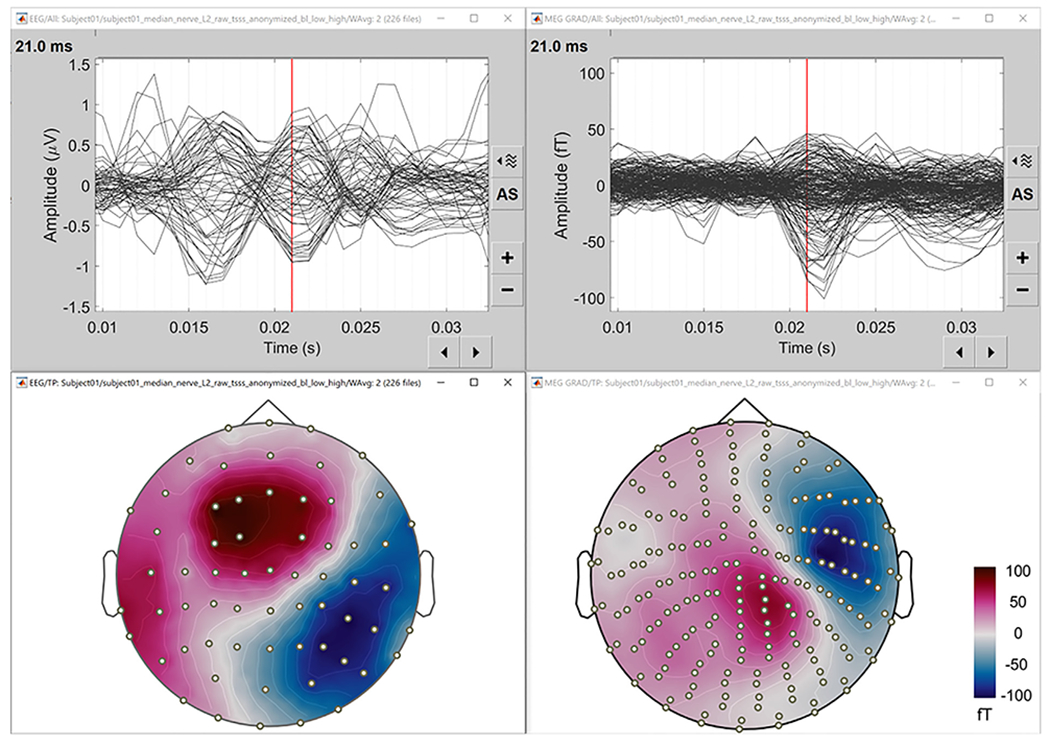
Preprocessed EEG/MEG data. Left: EEG, right: MEG. The top images are the butterfly plots; the bottom show the sensor topographies of the averaged potential at *t* = 21 ms.

The MEEG data were preprocessed as follows. First, a baseline correction was performed, data were then filtered between 20 Hz and 250 Hz (Buchner et al., 1995; Vorwerk et al., 2018). The EEG was referenced to common average reference. While all MEG channels had good data quality, a few segments of the EEG had to be rejected after visual inspection. Finally, a time-locked average of the trials was computed from 226 clean epochs. A butterfly plot and the peak topography at 21 ms of the resulting data are shown in Fig. 8. For more details, we refer readers to the online tutorial.

#### Sensor registration:

**Fig. 9.**
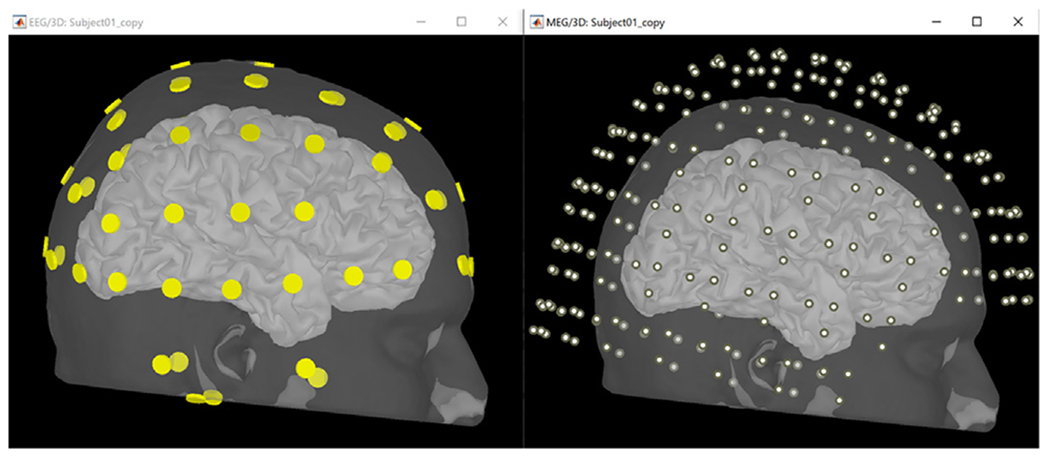
Visualization of the head model with aligned sensors. Left are the EEG electrodes on the scalp, right shows the MEG sensors around the head. The cortical surface on which the source space is defined is displayed in both images.

The sensor positions were aligned with the head model using standard Brainstorm functions. For MEG, the positions of the sensors were available with the recordings; for EEG electrodes, their positions were digitized as explained above and then co-registered to the head shape within Brainstorm. Figs. 8 and 9 show the locations of the 64 EEG electrodes and 306 MEG sensors respectively.

#### Source grid definition:

**Fig. 10.**
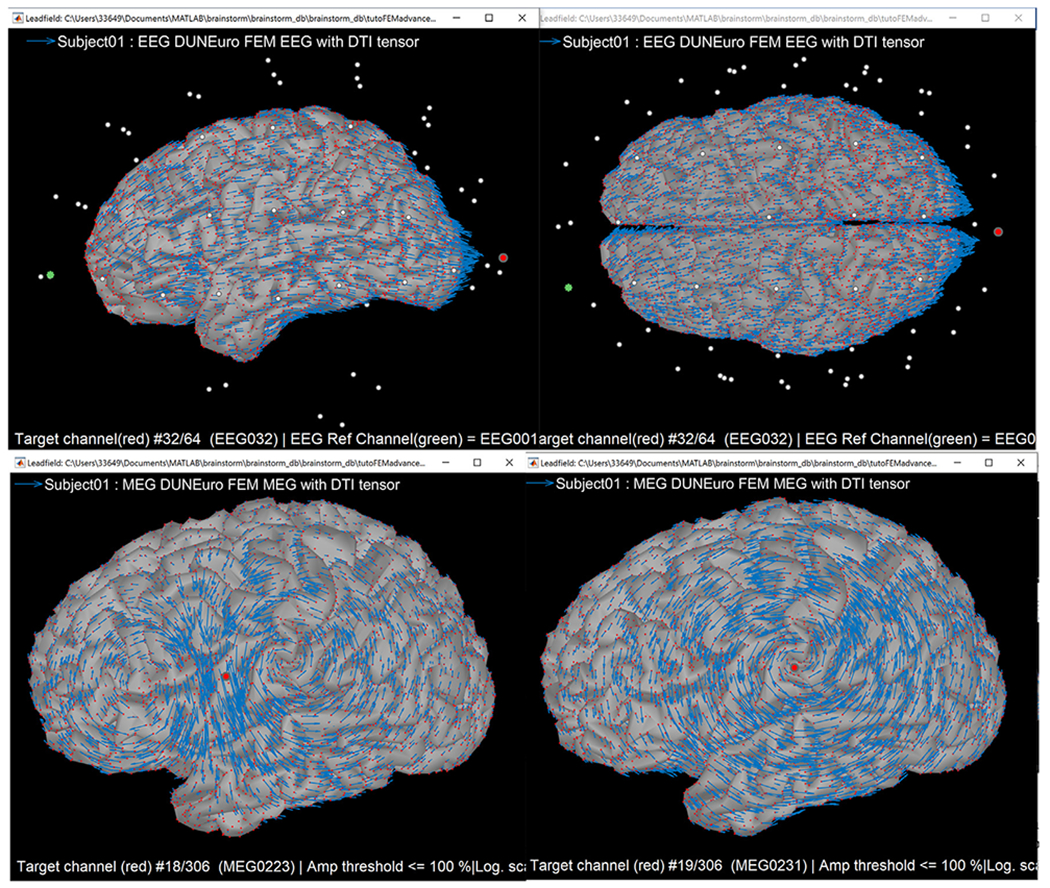
Visualization of cortical leadfield vectors from Brainstorm. Leadfield vectors show the orientation of sources with maximum sensitivity for the sensor type and locations shown. Top: FEM leadfield vectors for a selected pair of channels for the EEG lead FP1 in green and Oz in red. Bottom: The MEG leadfield for one sensor obtained with FEM, left is for a gradiometer, and right for a magnetometer. The small red dots on the cortex are the locations of the dipole sources.

The source space is a set of dipoles defined on the cortical surface and used for the forward computation. In this example, the cortex model was generated using SimNibs. Figs. 9 and 10 show views of the source space comprising ~15,000 cortical dipoles.

#### DUNEuro leadfield computation:

The final step of the FEM pipeline is computation of the leadfield matrix. In this example, the MEEG forward model was computed using DUNEuro’s standard continuous Galerkin FEM with the Venant source model with default parameters. Brainstorm’s built-in functions ensure that all dipole locations were positioned in the gray matter compartment to fulfill the Venant condition (Medani et al., 2015; Vorwerk et al., 2014, 2019a).

#### Leadfield visualization:

The images in Fig. 10 show distributions of the leadfield for an EEG (Top) electrode pair (colored red and green), and a single MEG (bottom) sensor in red, gradiometer on the left, and magnetometer on the right.

### 3.4. Inverse modeling and source localization

**Fig. 11.**
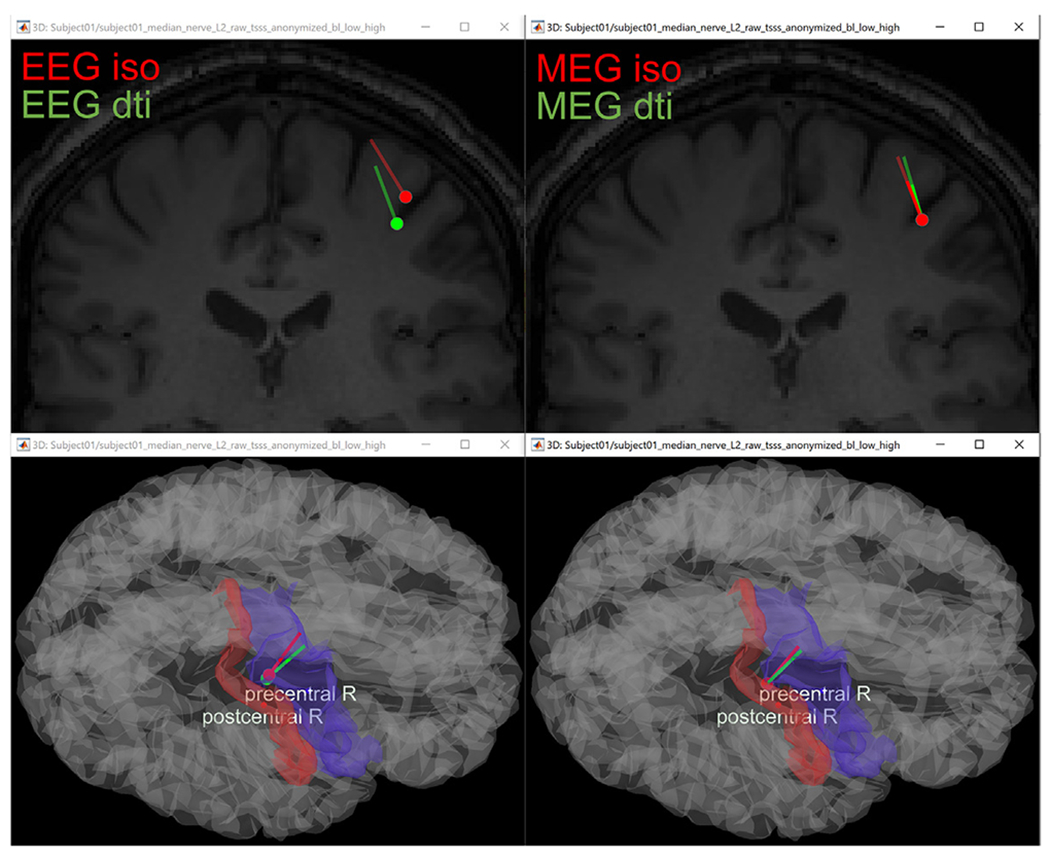
Reconstructed source of the P20 component and its location within the source space displayed on the MRI (top) and cortical surface (bottom). Left: the EEG reconstructed dipoles. Right: the reconstructed dipoles from MEG. Green (red) is the result computed using the head model with anisotropic (isotropic) white matter modeling.

Once the FEM forward model was computed, source localization of the SEP/SEF data was performed using the dipole scanning method (Generalized Least Squares solution, filtered for above-threshold goodness of fit. The detailed parameters and steps are explained in the online tutorial^22^). Fig. 11 shows the localized dipoles in the brain. The response of interest is the well-known P20/N20 component which is generated in Brodmann area 3b. The generators in this area are mainly superficial focal dipoles that are mostly tangentially oriented. Dipoles are reconstructed in primary somatosensory cortex in the post-central sulcal wall with predominantly tangential orientations, which reproduces findings of (Antonakakis et al., 2019; Aydin et al., 2014; Buchner et al., 1995). In this example, we found a localization difference of 4 mm between the EEG reconstructed sources when using the head models with isotropic versus anisotropic WM conductivity modeling. We did not see a significant localization difference for the MEG case. However, we observed a difference in the amplitude of the reconstructed dipole in both modalities (EEG and MEG); the magnitude in the anisotropic case is lower than the isotropic case. These results are in concordance with previous studies that found that conductivity uncertainties for white matter have little influence on source localization, but a strong influence on the strength and orientation of the reconstructed source (Güllmar et al., 2010; Vorwerk et al., 2019a). We also evaluated differences in source orientation, we found an angle of 25° between the EEG reconstructed sources when using the head models with isotropic versus anisotropic WM conductivity modeling. On the MEG side, the orientation difference is less significant (see Fig. 11).

### 3.5. Concordance with the other forward models

**Fig. 12.**
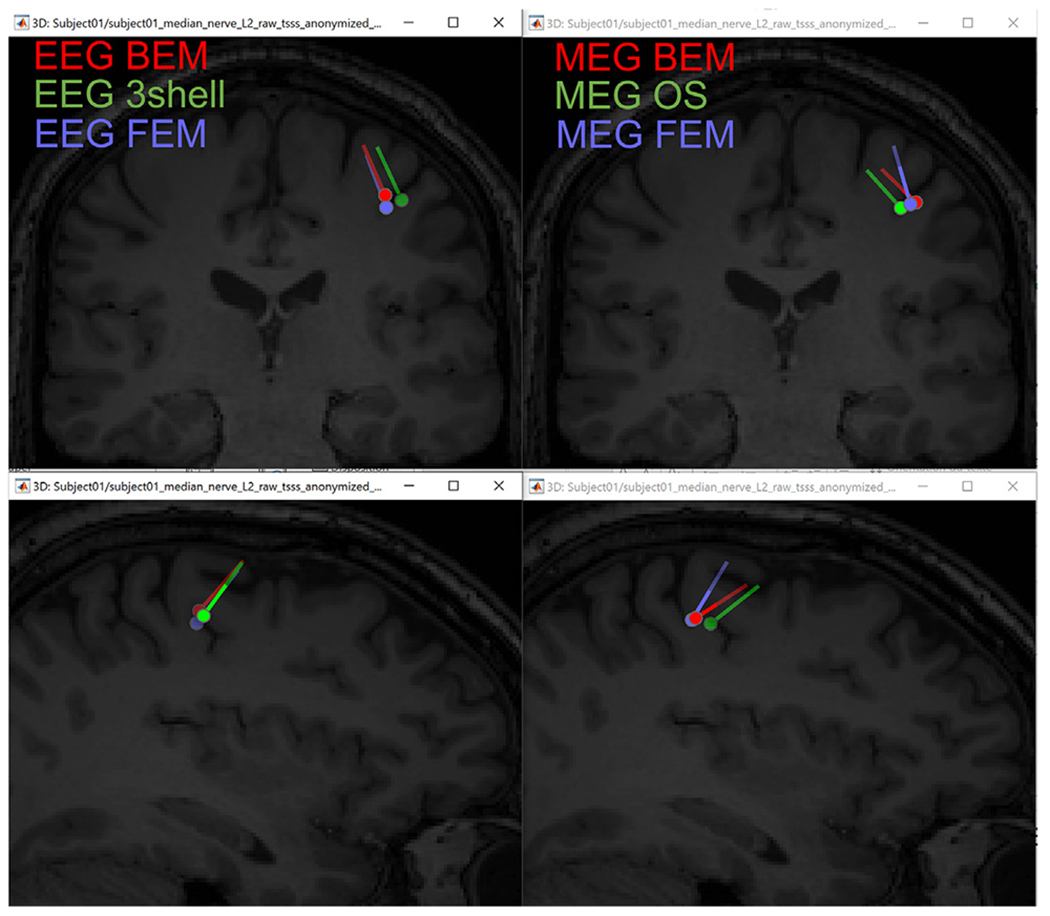
Reconstructed source of the P20/N20 component and its location within the source space displayed on the MRI (top: coronal; lower: sagittal). Left are the EEG-based dipoles. Right, are the dipoles from MEG. Green dipoles were computed from the analytical methods (overlapping spheres for MEG and 3shell for EEG), red from the BEM, and purple from the FEM.

To explore concordance of the FEM results with those from other forward methods already implemented within Brainstorm, we computed the leadfield using both the BEM with OpenMEEG (Gramfort et al., 2011) and the analytical spherical model for the same subject. For the BEM, first we constructed the head model using the standard BEM surface generator from Brainstorm; this model includes the inner skull, outer skull, and scalp. We performed the BEM computation using the default parameters. We also computed the leadfield using analytical solutions, respectively the 3-shell approach for EEG (Zhang, 1995) and the overlapping spheres approach for MEG (Huang et al., 1999), using default parameters in both cases. For each leadfield model we ran dipole scans using the same parameters as those used for the FEM with isotropic white matter modeling in the previous section. Fig. 12 shows the resulting dipole fits.

We observe that the analytical and BEM methods lead to dipoles sources located in the same area and with similar orientation and amplitude range when compared to those obtained with the FEM method. The maximum distance between these dipoles is 6.5 mm, with a 20° difference in orientation. These three approaches (BEM, FEM, and the analytical methods) are based on different theoretical approximations and implemented through independent functions; however, they all lead to comparable results.

Our goal here is to illustrate an application example rather than perform comparative evaluation of accuracy between these methods, a topic that has been addressed elsewhere (Hallez et al., 2007; Haueisen et al., 2002; Piastra et al., 2018; Vorwerk et al., 2019a; 2014, 2012; Wolters et al., 2006). Furthermore, we performed qualitative comparisons of the Brainstorm-DUNEuro forward results versus the other methods (analytical methods and BEM) and the obtained results can be viewed in the Brainstorm online tutorials.^23^

## 4. Conclusion

We have described the new FEM pipeline for realistic electromagnetic modeling of e-phys data that has been integrated into Brainstorm, a software environment widely used for neuroimaging data analysis. This new pipeline handles all steps, from processing MRI data for individual and realistic head model construction to accurate FEM leadfield computations and advanced visualization tools. Traditionally, the generation of a valid volumetric mesh from the MRI data, while not as complicated to follow as the forward modeling **per se**, has usually been regarded as tricky and cumbersome. This development allows integration of the entire workflow within the Brainstorm user interface, so that users do not have to combine the outputs of several independent tools. To show the practical use of this pipeline, we included the end-to-end example above, from volume conductor construction to source localization in a somatosensory stimulation MEEG experiment.

The accurate head model mesh is generated from MRI data by integrating modern tools including SimNibs, brain2mesh, fieldtrip, iso2mesh, and Roast. The anisotropic conductivity of white matter is computed from the DWI data using the Effective Medium Approach principle. FEM computation is performed using the DUNEuro library that incorporates modern FEM discretization methods and source models. The MEEG processing and source localization processes are performed within Brainstorm. At each step of this process, advanced visualization options are available; users can display the FEM head models, conductivity tensors, and leadfields. Different modalities and solutions from different models can be overlaid in the same figure, aiding with the visual inspection and comparison of results. All steps can be performed either from the Brainstorm graphical interface or scripting.

We also compared source localization results for the somatosensory EEG and MEG datasets using the FEM with previously validated methods (BEM and analytical methods). Overall, the FEM, the BEM and the analytical results show strong concordance among the methods and software implementations.

Documentation, full step-by-step tutorials, and complete dataset examples are available on the Brainstorm website (https://neuroimage.usc.edu/brainstorm). Users can reproduce the pipeline presented here by following the online tutorials.

Our primary purpose in developing this tool was to provide the neuroscience community with relatively easy-to-use advanced computational electromagnetic modeling tools that do not require advanced programming skills or expert knowledge of FEM modeling.

In addition to the implementation itself, we have added measures towards long-term maintainability. This issue has two important aspects: (i) current code adapting to future changes in MATLAB and operating systems, and (ii) future features and versions of DUNEuro being added to DUNEuro-Brainstorm. To address the first issues, users can follow a step-by-step tutorial to download all the required dependencies and a compilation script that automatically generates the binary executable applications for each platform. These tools are stored in a freely accessible repository named bst-duneuro,^24^ which is part of the brainstorm-tools organization and maintained directly by the Brainstorm team. Additionally, the C++ framework (compiler, linker, standard libraries, etc.) is strongly backwards compatible, thus the forward compatibility of the script is relatively secure. Lastly, to make DUNEuro-Brainstorm more easily compatible with future developments of DUNEuro, we included the compilation script within the “official” build system of DUNE^25^ and DUNEuro. We compile DUNEuro only through this interface, therefore future changes in DUNEuro will be made automatically compatible with our compilation script.

Although not presented here, this FEM software can also be used for modeling of leadfields for intracranial modalities including SEEG and ECOG (Medani et al., 2022). Through the principle of reciprocity, we know that the sensitivity distribution in the detection of bioelectric signals, is the same distribution that governs the energy distribution of an electromagnetic field applied during an electromagnetic stimulation (Malmivuo and Plonsey, 2012). For this reason, it has been shown (Gross et al., 2021) how this pipeline can also be readily extended to allow computation for trans- and intra-cranial electric and magnetic stimulation research (Medani et al., 2022).

In the future, we plan to support options already implemented in DUNEuro from within this interface, such as the discontinuous Galerkin (Engwer et al., 2017; Nüßing et al., 2016; Piastra et al., 2018) and unfitted FEM methods based on level set tissue surface segmentations (Erdbrügger et al., 2022; Schrader et al., 2021). We note that unfitted FEM (Engwer et al., 2017; Erdbrügger et al., 2022; Nüßing et al., 2016; Piastra et al., 2018; Schrader et al., 2021; Vallaghé and Papadopoulo, 2010) does not use the detailed tessellation of the head domain but relies instead on an implicit description of the tissue layers via the level-set functions. Here we use only the more widely studied fitted methods.

## Data Availability

The code and the data used in this paper are freely available from the Brainstorm website.

## Footnotes

1 Commercial software packages are not included in this paper.

2 https://www.mrt.uni-jena.de/simbio.

3 https://dune-project.org/.

4 GNU Project Lesser General Public License.

5 https://www.medizin.uni-muenster.de/duneuro.

6 https://neuroimage.usc.edu/brainstorm.

7 https://neuroimage.usc.edu/brainstorm/Tutorials/SourceEstimation.

8 https://neuroimage.usc.edu/brainstorm/Tutorials/TutBem.

9 https://neuroimage.usc.edu/brainstorm/Tutorials/FemMesh.

10 The FEM mesh plugins are implemented within the *process_fem_mesh.m*.

11 http://www.fieldtriptoolbox.org/reference/ft_volumesegment/.

12 http://www.fieldtriptoolbox.org/reference/ft_prepare_mesh/.

13 https://neuroimage.usc.edu/brainstorm/Tutorials/FemMesh.

14 *dwi2dti* refers to the Brainstom’s function *process_dwi2dti.m*

15 http://brainsuite.org/.

16 *dti2cond* refers to the Brainstom’s function *process_fem_tensor.m*

17 https://neuroimage.usc.edu/brainstorm/Tutorials/TutUserProcess.

18 https://neuroimage.usc.edu/brainstorm/Tutorials/FemMesh.

19 https://github.com/brainstorm-tools/bst-DUNEuro.

20 https://neuroimage.usc.edu/brainstorm/Tutorials/Scripting.

21 https://neuroimage.usc.edu/brainstorm/Tutorials/FemMedianNerve.

22 https://neuroimage.usc.edu/brainstorm/Tutorials/FemMedianNerve.

23 https://neuroimage.usc.edu/brainstorm/Tutorials/ReviewForward.

24 https://github.com/brainstorm-tools/bst-duneuro.

25 DUNE is a framework for solving partial differential equations on top of which DUNEuro is built.
